# The RNA‐helicase DDX21 upregulates CEP55 expression and promotes neuroblastoma

**DOI:** 10.1002/1878-0261.12906

**Published:** 2021-02-26

**Authors:** Vina Putra, Amy J. Hulme, Andrew E. Tee, Jane Q.J. Sun, Bernard Atmadibrata, Nicholas Ho, Jingwei Chen, Jixuan Gao, Murray D. Norris, Michelle Haber, Maria Kavallaris, Michelle J. Henderson, Joshua McCarroll, Toby Trahair, Tao Liu, Pei Y. Liu

**Affiliations:** ^1^ Children’s Cancer Institute Lowy Cancer Research Centre UNSW Sydney Kensington NSW Australia; ^2^ University of New South Wales Centre for Childhood Cancer Research Sydney NSW Australia; ^3^ ARC Centre of Excellence in Convergent Bio‐Nano Science and Technology Australian Centre for Nanomedicine UNSW Sydney Kensington NSW Australia; ^4^ School of Women’s and Children’s Health Faculty of Medicine UNSW Sydney Kensington NSW Australia

**Keywords:** amplification, CEP55, DDX21, *MYCN*, neuroblastoma, N‐Myc

## Abstract

Approximately 25% of human neuroblastoma is caused by amplification of the *MYCN* oncogene, which leads to overexpression of N‐Myc oncoprotein. The survival rate for this patient subtype is <50%. Here, we show that N‐Myc protein bound to the DEAD‐box RNA helicase *DDX21* gene promoter and upregulated DDX21 mRNA and protein expression. Genome‐wide differential gene expression studies identified centrosomal protein CEP55 as one of the genes most dramatically downregulated after DDX21 knockdown in *MYCN*‐amplified neuroblastoma cells. Knocking down DDX21 or CEP55 reduced neuroblastoma cell cytoskeleton stability and cell proliferation and all but abolished clonogenic capacity. Importantly, DDX21 knockdown initially induced tumor regression in neuroblastoma‐bearing mice and suppressed tumor progression. In human neuroblastoma tissues, a high level of DDX21 expression correlated with a high level of N‐Myc expression and with CEP55 expression, and independently predicted poor patient prognosis. Taken together, our data show that DDX21 induces CEP55 expression, *MYCN*‐amplified neuroblastoma cell proliferation, and tumorigenesis, and that DDX21 and CEP55 are valid therapeutic targets for the treatment of *MYCN*‐amplified neuroblastoma.

## Graphical abstract

The *MYCN* oncogene encodes an ‘undruggable’ protein N‐Myc. Here, we show that N‐Myc protein binds to the DDX21 gene promoter, leading to increased CEP55 expression, neuroblastoma cell cytoskeletal stability, and cell proliferation. Knocking down DDX21 suppressed tumor progression in neuroblastoma‐bearing mice. Our data demonstrate that DDX21 and CEP55 are valid therapeutic targets for the treatment of *MYCN*‐amplified neuroblastoma.

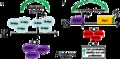

**Abbreviations**
CEP55: centrosomal protein 55
DDX21: DEAD‐box RNA helicase 21
DDX3: DEAD‐box RNA helicase 3
DDX41: DEAD‐box RNA helicase 41
DOX: doxycycline
E‐box: enhancer box
shRNA: short hairpin RNA
siRNA: small interfering RNA

## 1 Introduction

Neuroblastoma is the most common solid extracranial tumor in children and responsible for 15% of all childhood cancer deaths. Amplification of the *MYCN* oncogene occurs in ~ 25% of neuroblastoma patients. Despite multimodal therapy including chemotherapy, surgery, radiotherapy, myeloablative therapy with autologous transplant, and immunotherapy, survival rates for *MYCN*‐amplified neuroblastoma patients remain poor [1, 2].

*MYCN* gene amplification leads to overexpression of N‐Myc oncoprotein and neuroblastoma tumorigenesis [2, 3]. Through its basic helix–loop–helix zipper domain, N‐Myc heterodimerizes with another basic helix–loop–helix zipper protein Max, forming a transcription factor complex that binds to specific Myc‐responsive enhancer box (E‐box) DNA motifs. This binding activates the transcription of target genes involved in various cellular processes, including cell growth, proliferation, metabolism, apoptosis, and differentiation [4, 5]. The N‐Myc oncoprotein is considered ‘undruggable’ because it has no active sites for ligand binding by conventional small molecule drug‐like compounds and has not been crystalized [6, 7]. Thus, rather than targeting N‐Myc directly, targeting downstream factors vital for N‐Myc's oncogenic effects is an attractive approach for the therapy of *MYCN*‐amplified neuroblastoma.

RNA helicases are categorized into six superfamilies, of which DEAD‐box helicases are the largest group and are characterized by nine conserved motifs for ATP binding, hydrolysis, intramolecular rearrangements, and RNA interactions [8, 9]. DEAD‐box helicases are multifunctional, being involved in gene transcription, pre‐mRNA splicing, microRNA processing, and RNA metabolism, while aberrant expression of these critical regulators is linked to neurodegenerative diseases, dysregulation of innate immune responses, and cancers [8, 9, 10]. Small molecule inhibitors have been successfully developed to target the DEAD‐box RNA helicases eukaryotic initiation factor‐4A, DEAD‐box RNA helicase 3 (DDX3), and DDX41 [11, 12, 13, 14], with the DDX3 inhibitor RK‐33 now in clinical trials for lung cancer patients. DEAD‐box RNA helicases therefore represent promising therapeutic targets.

DEAD‐box RNA helicase DDX21 binds to target gene promoters and facilitates the release of the positive transcription elongation factor b (P‐TEFb) to induce target gene transcription [15]. DDX21 is overexpressed in human breast and colon cancer tissues [16, 17] and contributes to leukemogenesis and breast cancer tumorigenesis [16, 18]. In this study, bioinformatics analysis identified Myc‐responsive E‐boxes at the *DDX21* gene promoter. N‐Myc upregulated *DDX21* gene transcription, which subsequently upregulated *CEP55* transcriptional elongation and resulted in increased neuroblastoma cell proliferation *in vitro* and tumor progression in mice. In addition, high DDX21 expression correlated with high N‐Myc and centrosomal protein 55 (CEP55) expression and predicted poor patient prognosis in human neuroblastoma.

## 2 Materials and methods

### 2.1 Cell culture

Cell lines were validated and maintained as previously described [19]. Briefly, BE(2)‐C and SHEP‐21N neuroblastoma cells were maintained in Dulbecco's Modified Eagle's medium (DMEM) containing 10% FBS. Kelly neuroblastoma cells were grown in Roswell Park Memorial Institute Medium (RPMI) 1640 containing 10% FBS. BE(2)‐C cells were provided by Barbara Spengler (Fordham University, NY, USA). Kelly cells were purchased from the European Collection of Cell Cultures (Sigma, St. Louise, MO, USA), and SHEP‐21N cells were provided by S. Cohn (The University of Chicago, USA). All cell lines were authenticated by small tandem repeat profiling (CellBank Australia or Garvan Institute of Medical Research, Sydney, Australia).

### 2.2 Establishment of neuroblastoma cell lines stably expressing DOX‐inducible shRNA

The lentiviral construct, DOX‐inducible GFP‐shRNA FH1tUTG, was provided by M. Herold [20] and used to produce control or DDX21 short hairpin RNA (shRNA) expression constructs as described previously [21]. The control shRNA and DDX21 shRNA‐1 and DDX21 shRNA‐2 target sequences were 5′‐GCACTACCAGAGCTAACTCAGATAGTACT‐3′, 5′‐CGCTCCTTGATCAACTCAAAT‐3′, and 5′‐GGAGACACTGCGAAAGCAAAC‐3′, respectively. Forward and reverse control shRNA, DDX21 shRNA‐1, and DDX21 shRNA‐2 oligonucleotides were inserted into the DOX‐inducible GFP‐shRNA FH1tUTG vector. HEK293T cells were then transfected with these constructs, viral medium was harvested and used to transduce BE(2)‐C and Kelly neuroblastoma cells in the presence of polybrene (8 µg·mL^−1^) (Santa Cruz Biotechnology, Santa Cruz, CA, USA) for 72 h. Cells expressing high GFP were sorted with BD FACS Jazz™ II Cell Sorter (BD Biosciences, Franklin Lakes, NJ, USA). BE(2)‐C and Kelly cells containing the shRNAs were treated with 2 µg·mL^−1^ of DOX (Sigma) daily to activate shRNA expression *in vitro*.

### 2.3 Luciferase assay

Luciferase assays were used to examine the activity of *DDX21* gene promoter modulated by N‐Myc and *CEP55* gene promoter modulated by DDX21. *DDX21* gene promoter region from −870 bp upstream to +111 bp downstream and *CEP55* gene promoter region from −884 bp upstream to +165 bp downstream of the *CEP55* transcription start site were custom‐cloned into pLightSwitch_Prom by Switchgear (Active Motif, Carlsbad, CA, USA). BE(2)‐C cells were transfected with control siRNA, N‐Myc siRNA‐1, or N‐Myc siRNA‐2 using Lipofectamine 2000 (Invitrogen, Carlsbad, CA, USA) for 14 h; subsequently, the cells were transfected with the empty vector control or *DDX21* gene promoter construct using Lipofectamine LTX with PLUS Reagent (Invitrogen) for 30 h, followed by luciferase assays. The empty vector control or *CEP55* gene promoter construct together with the Cypridina TK control construct was transfected into the BE(2)‐C cells containing DOX‐inducible control or DDX21 shRNA using Lipofectamine LTX with PLUS Reagent, for 24 h, then treated with vehicle control or DOX for 30 h followed by luciferase assays. Luciferase activity was quantified using the LightSwitch Dual‐Luciferase Assay System (Active Motif), and RenSP reporter signal was normalized by the Cypridina reporter signal. Normalized RenSP reporter signal was then calculated as fold change relative to the empty vector construct and vehicle control treatment condition as previously described [21].

### 2.4 Animal studies

Animal studies were performed with approval from the Animal Care and Ethics Committee of UNSW Sydney (Approval #: 17/35B), Australia, and in agreement with institutional guidelines. Four‐ to five‐week‐old female BALB/c nude mice were anesthetized and subcutaneously injected with 2 × 10^6^ DOX‐inducible control shRNA or DDX21 shRNA‐1 BE(2)‐C cells (in serum‐free DMEM cell culture medium), or 6 × 10^6^ DDX21 shRNA‐2 Kelly cells (in serum‐free RPMI cell culture medium) into the right flank. When the tumors reached 0.05 cm^3^, the mice were randomized into two groups and fed with food containing vehicle control or 600 mg·kg^−1^ of DOX (Meat Free Rat and Mouse Finished Diet, Specialty Feeds Pty. Ltd., Glen Forrest, WA, Australia). Tumors were measured every second day, and their volumes were calculated using the formula (length × width × height)/2. Once the tumors reached 1.0 cm^3^, the mice were sacrificed and tumors were harvested as previously reported [21].

### 2.5 siRNA transfection

Neuroblastoma cells were transfected with a validated negative control (All Star; Qiagen, Hilden, Germany), and N‐Myc or CEP55 siRNAs (Qiagen) using Lipofectamine 2000 (Invitrogen) as previously described [21], followed by RNA or protein extraction 48 h after transfection. The target siRNA sequences were as follows: 5′‐CGTGCCGGAGTTGGTAAAGAA‐3′ (siRNA‐1) and 5′‐TCCAGCGAGCTGATCCTCAAA‐3′ (siRNA‐2) for N‐Myc; and 5′‐CTCCAGCATGCTAGTGAATCA‐3′ (siRNA‐1) and 5′‐ATCAGCTGTTGTATTCACAAA‐3′ (siRNA‐2) for CEP55.

### 2.6 Real‐time reverse transcription PCR (RT‐PCR)

RT‐PCR was performed using Power SYBR Green Master Mix (Thermo Fisher Scientific, Waltham, MA, USA) and gene‐specific oligonucleotides on Applied Biosystems 7900 or QuantStudio™ (Applied Biosystems, Grand Island, NY, USA), and quantified using the comparative threshold cycle (△△Ct) method [22], relative to the housekeeping gene actin or B2M as previously reported [21]. The RT‐PCR oligonucleotides were manufactured by Sigma, and their sequences were as follows: 5′‐CGACCACAAGGCCCTCAGTA‐3′ (forward) and 5′‐CAGCCTTGGTGTTGGAGGAG‐3′ (reverse) for N‐Myc; 5′‐GCTCTTATCTCAGGTCCAGTTTCTTT‐3′ (forward) and 5′‐GAGCTACCCTTGTTTGTTCTTCTTG‐3′ (reverse) for CEP55; 5′‐GGCGAGGAGATTGATTCCAA −3′ (forward) and 5′‐CAAACACCCAGCTTTCCTTTG‐3′ (reverse) for DDX21; 5′‐GCTGTGCTCGCGCTACTCT‐3′ (forward) and 5′‐TGAATCTTTGGAGTACGCTGGAT‐3′ (reverse) for B2M; and 5′‐AGGCCAACCGCGAGAAG‐3′ (forward) and 5′‐ACAGCCTGGATAGCAACGTACA‐3′ (reverse) for actin.

### 2.7 Western blotting

Western blotting was performed as previously described [21]. The primary antibodies used were as follows: rabbit anti‐DDX21 (1 : 500; Catalogue number A300‐629A; Bethyl Laboratories, Montgomery, TX, USA), rabbit anti‐CEP55 (1 : 500; Catalogue number 81693S; Cell Signaling Technology, Danvers, MA, USA) or mouse anti‐N‐Myc (1 : 1000; Catalogue number sc‐53993; Santa Cruz Biotechnology). The secondary antibodies used were as follows: goat anti‐rabbit (Catalogue number 12‐348) or goat anti‐mouse (Catalogue number 12‐349) (1 : 10 000; both from MERCK, Burlington, MA, USA). The anti‐actin antibody (1 : 15 000; Catalogue number A3853; Sigma) was used as loading control.

### 2.8 Differential gene expression analysis by Affymetrix microarray

BE(2)‐C cells stably expressing the DOX‐inducible control or DDX21 shRNAs were treated with vehicle control or DOX for 32 h followed by RNA extraction with RNeasy Mini Kit (Qiagen). Affymetrix microarrays (Affymetrix, Santa Clara, CA, USA) were performed to examine genome‐wide differential gene expression after DDX21 knockdown. The microarray data were analyzed in R (http://www.r‐project.org/) with bioconductor package (http://www.bioconductor.org/) and normalized with RMA normalization method (https://www.ncbi.nlm.nih.gov/pubmed/12925520) as previously described [21].

### 2.9 ChIP assays

ChIP assays were performed to assess N‐Myc protein enrichment at the *DDX21* gene promoter as previously described [23] with slight modification. Briefly, histone proteins and DNA in BE(2)‐C cells were cross‐linked by incubation with 1% formaldehyde in culture medium for 15 min. ChIP assays were performed with a mouse anti‐N‐Myc (Catalogue number sc‐53993; Santa Cruz Biotechnology) or a control mouse IgG (Catalogue number 31903; Invitrogen), followed by PCR analysis with primers targeting the negative control region or the core promoter region of the *DDX21* gene. The primer sequences used were as follows: 5′‐CTGCATTCATGCTTGCTCTC‐3′ (sense) and 5′‐GAGCCAGGAGCCTTGATTTA‐3′ (antisense) for the remote negative control region of the *DDX21* gene; 5′‐GCAATTCCTTGCGGTTCTAC‐3′ (sense) and 5′‐AAGCAGGAAGCACAGGAAAA‐3′ (antisense) for the *DDX21* gene promoter containing the noncanonical E‐box at −93 to −98 bp upstream of the *DDX21* transcriptional start site; and 5′‐CTACGGGAAGTGACGAGAGC‐3′ (sense) and 5′‐GCGTCACTACGGAGTTTTCC‐3′ (antisense) for the *DDX21* gene promoter containing the noncanonical E‐box at +12 to +17 bp downstream of the *DDX21* gene transcriptional start site.

To analyze DDX21 protein binding to the *CEP55* gene promoter, histone proteins and DNA in BE(2)‐C cells were cross‐linked with Diagenode ChIP cross‐link Gold, followed by incubation with 1% formaldehyde. ChIP assays were performed with a rabbit anti‐DDX21 (Catalogue number NBP1‐83310; Novus Biologicals, Littleton, CO, USA) or a control rabbit IgG (Catalogue number 31235; Invitrogen), followed by PCR analysis with primers targeting the negative control region or the *CEP55* gene core promoter region. The PCR primer sequences used were as follows: 5′‐ATTCGCTCAATCACTGTGGTTCT‐3′ (sense) and 5′‐TGAGAGTGATTCTTTGGTTGGTATCT‐3′ (antisense) for the remote negative control region of the *CEP55* gene; and 5′‐AGGTTTACTCTCCGCCCTTC‐3′ (sense) and 5′‐GCTTCCGGGAGTTTGAATC‐3′ (antisense) for the *CEP55* gene core promoter.

To analyze phosphorylated S2 Pol II binding to the *CEP55*, *RPLP2,* and *β‐actin* gene promoters and transcriptional termination sites, DOX‐inducible DDX21 shRNA‐1 BE(2)‐C cells were treated with vehicle control or DOX for 48 h, followed by ChIP assays as described above with a rabbit anti‐phosphorylated S2 Pol II (Catalogue number ab5095; Abcam, Cambridge, UK) or a control rabbit IgG (Invitrogen) antibody. The PCR primer sequences used were as follows: 5′‐TGGCCACACACAATGTTTTC‐3′ (sense) and 5′‐TGAGAGGGCTACATGGGTTT‐3′ (antisense) for *CEP55* 3′‐UTR; 5′‐CACCAAGGAGTCAAGGCGAG‐3′ (sense) and 5′‐CTCAACCTTTGCCAGCGAAC‐3′ (antisense) for the *RPLP2* gene core promoter; 5′‐ATGGATGCAGGAAGTGAGCC‐3′ (sense) and 5′‐AAGCCTGAGGAGTGATTGCC‐3′ (antisense) for the *RPLP2* 3′‐UTR; 5′‐CTCAATCTCGCTCTCGCTCT‐3′ (sense) and 5′‐TCGAGCCATAAAAGGCAACT‐3′ (antisense) for the *β‐actin* gene core promoter; and 5′‐CACAGGGGAGGTGATAGCAT‐3′ (sense) and 5′‐CTCAAGTTGGGGGACAAAAA‐3′ (antisense) for the *β‐actin* 3′‐UTR.

Fold enrichment of N‐Myc protein at the *DDX21* gene core promoter or DDX21 protein at the *CEP55* gene core promoter was calculated as previously described [23] by dividing the Ct threshold obtained from PCR products of samples pulled down by N‐Myc or DDX21 antibody by the Ct threshold obtained from PCR products of samples pulled down by the control antibody.

### 2.10 Immunocytochemistry

BE(2)‐C and Kelly cells were transfected with control siRNA, CEP55 siRNA‐1, or CEP55 siRNA‐2 for 72 h in Lab‐Tek™ Chamber slides (Thermo Fisher Scientific). In separate experiments, BE(2)‐C or Kelly cells containing DOX‐inducible control shRNA, DDX21 shRNA‐1, or DDX21 shRNA‐2 were plated in Lab‐Tek™ Chamber slides for 24 h, then treated with vehicle control or DOX every 24 h for a total of 72 h. Cells were then fixed in 4% paraformaldehyde for 15 min, permeabilized with 0.2% Triton X‐100, blocked with 10% FBS, and probed with Alexa Fluor 647 phalloidin (Thermo Fisher Scientific), or rabbit anti‐α‐tubulin antibody (Abcam) and Alexa Fluor 594‐conjugated goat anti‐rabbit antibody (Thermo Fisher Scientific) and DAPI (Vector Laboratories, Burlingame, CA, USA).

Images were taken under a confocal fluorescence microscope (Leica TCS SP8, Wetzlar, Germany), and α‐tubulin filaments were quantified by dividing the area of α‐tubulin filaments by the area of the nucleus for each cell. These analyses were done using a custom‐built MATLAB code, which first converted the images into binary images. The binary mask was cleaned by morphological operations using the MATLAB built‐in operation of structuring element, dilation, and erosion [24].

### 2.11 Alamar blue and BrdU assays

BE(2)‐C or Kelly cells were transfected with siRNAs in 96‐well plates. In separate experiments, BE(2)‐C or Kelly cells containing DOX‐inducible control shRNA, DDX21 shRNA‐1, or DDX21 shRNA‐2 were plated in 96‐well plates the day before and treated with vehicle control or DOX every 24 h. Alamar blue (Invitrogen) or BrdU was added to the cells 90 h after transfection or 72 h after DOX treatment and read on a microplate reader (Victor3; PerkinElmer, Waltham, MA) at 570/595 nm or 492/690 nm, respectively. Results were calculated as described previously [21].

### 2.12 CEP55 overexpression

CEP55 open reading frame was custom‐cloned into a lentiviral construct by VectorBuilder. Empty vector control or CEP55 overexpression construct was cotransfected with three helper plasmids into HEK293T cells using Lipofectamine 3000 (Invitrogen). Viral medium was harvested and used to transduce BE(2)‐C cells containing DOX‐inducible control shRNA, DDX21 shRNA‐1, or DDX21 shRNA‐2 in the presence of polybrene (8 µg·mL^−1^).

### 2.13 Cell cycle analysis

BE(2)‐C and Kelly cells were transfected with control siRNA, CEP55 siRNA‐1, or CEP55 siRNA‐2 for 72 h, followed by propidium iodide staining and flow cytometry analysis. In separate experiments, BE(2)‐C and Kelly cells containing DOX‐inducible control shRNA, DDX21 shRNA‐1, or DDX21 shRNA‐2 were treated with vehicle control or DOX every 24 h for a total of 72 h, followed by propidium iodide staining and flow cytometry analysis.

### 2.14 Statistical analysis

Every experiment was performed at least three times. Statistical significance was analyzed using two‐sided unpaired *t*‐test for two groups or ANOVA for multiple groups in graphpad prism 8 (GraphPad, San Diego, CA, USA) as previously described [21].

The Pearson correlation and Kaplan–Meier analyses in the graphpad prism program were used to examine the association between DDX21 and N‐Myc or DDX21 and CEP55 expression, and overall survival in human neuroblastoma patients, respectively, as previously described [21]. Multivariable Cox regression analysis in the IBM SPSS program was used to investigate the clinical significance of DDX21 and CEP55 in human neuroblastoma. The prognostic factors included in the analysis were as follows: age at diagnosis, INSS stage, and *MYCN* amplification status. Survival probabilities and hazard ratios (HRs) were calculated with 95% CIs as previously described [21].

## 3 Results

### 3.1 N‐Myc increases DDX21 expression by binding to the *DDX21* gene promoter

**Fig. 1:**
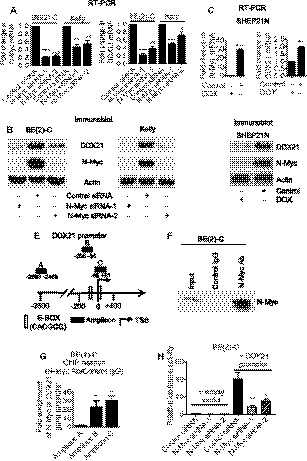
N‐Myc upregulates *DDX21* gene expression by binding to the *DDX21* gene promoter in neuroblastoma cells. (A–B) BE(2)‐C and Kelly cells were transfected with control siRNA, N‐Myc siRNA‐1, or N‐Myc siRNA‐2 for 48 h, followed by RT‐PCR (A) and western blot (B) analyses of N‐Myc and DDX21 mRNA and protein expression. (C–D) Tetracycline withdrawal‐inducible N‐Myc overexpression SHEP‐21N cells were treated with vehicle control or DOX for 48 h, followed by RT‐PCR (C) and western blot (D) analyses of N‐Myc and DDX21 mRNA and protein expression. (E) Schematic representation of the DDX21 gene promoter. TSS, transcription start site. (F–G) ChIP assays were performed with a control or an anti‐N‐Myc antibody (Ab), the immunoprecipitated protein was subjected to immunoblot analysis with an anti‐N‐Myc antibody (F), and the immunoprecipitated DNA was examined by PCR (G) with primers targeting a negative control region (Amplicon A) or the DDX21 gene promoter regions containing the E‐boxes (Amplicons B and C) in BE(2)‐C cells. Fold enrichment of N‐Myc at the DDX21 gene promoter regions was calculated as the difference in cycle thresholds obtained with the anti‐N‐Myc Ab and with the control Ab. (H) BE(2)‐C cells were transfected with control siRNA, N‐Myc siRNA‐1, or N‐Myc siRNA‐2 for 14 h, then transfected with empty vector control or *DDX21* gene promoter (−870 to +111 bp) in LightSwitch reporter construct for 30 h followed by luciferase assays. Luciferase activities were expressed as fold changes relative to empty vector construct. Error bars represented standard error. * indicates *P* < 0.05, **P < 0.01, and ***P < 0.001.

It is well established that N‐Myc binds to Myc‐responsive E‐box motifs and activates gene transcription [25]. By analyzing gene promoter sequence data from the National Centre for Biotechnology Information (NCBI), we found two noncanonical Myc‐responsive E‐boxes (CACGCG) at −93 to −98 bp upstream and +12 to +17 bp downstream of the *DDX21* gene transcriptional start site. Consequently, two *MYCN*‐amplified neuroblastoma cell lines, BE(2)‐C and Kelly, were transfected with a control small interfering RNA (siRNA) or two different N‐Myc siRNAs. RT‐PCR and western blot analyses revealed that both N‐Myc siRNAs successfully knocked down N‐Myc mRNA (Fig. 1A) and protein (Fig. 1B) expression and that DDX21 mRNA (Fig. 1A) and protein (Fig. 1B) levels were considerably decreased after N‐Myc knockdown. To further confirm the regulation of DDX21 expression by N‐Myc, we used doxycycline (DOX) withdrawal‐inducible N‐Myc overexpressing SHEP‐21N neuroblastoma cells. RT‐PCR and western blot analyses showed that removal of DOX resulted in N‐Myc mRNA (Fig. 1C) and protein (Fig. 1D) overexpression, and significantly increased DDX21 mRNA (Fig. 1C) and protein (Fig. 1D) expression.

To investigate whether N‐Myc binds to the *DDX21* gene promoter, chromatin immunoprecipitation (ChIP) assays were performed with a control or an anti‐N‐Myc antibody in BE(2)‐C cell lysates. The N‐Myc antibody enhanced the pull down of N‐Myc protein and the DDX21 proximal promoter region containing the noncanonical E‐box regions (Amplicons B and C) by ~ 20‐ to 30‐fold compared with the negative control (Amplicon A) (Fig. 1E–G). To further confirm the transcriptional control of DDX21 by N‐Myc, BE(2)‐C cells were transfected with control or two different N‐Myc siRNAs, followed by transfection with a LightSwitch luciferase reporter construct containing the *DDX21* gene promoter and luciferase assays. Correspondingly, knocking down N‐Myc significantly decreased the luciferase activity at the *DDX21* gene promoter (Fig. 1H).

In summary, these data suggest that N‐Myc upregulates DDX21 gene expression by binding to the *DDX21* gene promoter.

### 3.2 DDX21 binds to the *CEP55* gene promoter and upregulates CEP55 gene expression

**Fig. 2:**
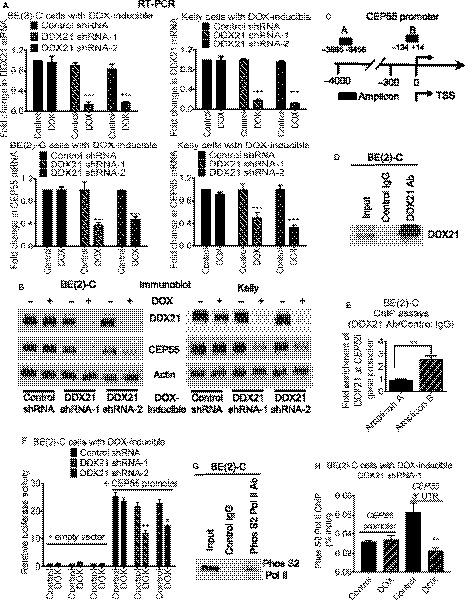
DDX21 upregulates CEP55 gene expression by binding to the *CEP55* gene promoter and promotes CEP55 transcriptional elongation. (A–B) DOX‐inducible control shRNA, DDX21 shRNA‐1, or DDX21 shRNA‐2 BE(2)‐C and Kelly cells were treated with vehicle control or DOX for 48 h, followed by RT‐PCR (A) or western blot (B) analyses of DDX21 and CEP55 mRNA and protein expression. (C) Schematic representation of the *CEP55* gene promoter. TSS, transcription start site. (D–E) ChIP assays were performed with a control or anti‐DDX21 antibody (Ab), the immunoprecipitated protein was subjected to immunoblot analysis with an anti‐DDX21 antibody (D), and the immunoprecipitated DNA was examined by PCR (E) with primers targeting the negative control region (Amplicons A) or the *CEP55* gene core promoter (Amplicons B) in BE(2)‐C cells. Fold enrichment of DDX21 at the *CEP55* gene promoter was calculated as the difference in cycle thresholds obtained with the anti‐DDX21 Ab and with the control Ab. (F) DOX‐inducible control shRNA, DDX21 shRNA‐1, or DDX21 shRNA‐2 BE(2)‐C cells were transfected with empty vector control or *CEP55* gene promoter (−884 to +165 bp) in LightSwitch reporter construct for 24 h, then treated with vehicle control or DOX for 30 h followed by luciferase assays. Luciferase activities were expressed as fold changes relative to control shRNA, DDX21 shRNA‐1, or DDX21 shRNA‐2 BE(2)‐C cells transfected with empty vector control and treated with vehicle control. (G–H) DOX‐inducible DDX21 shRNA‐1 BE(2)‐C cells were treated with vehicle control or DOX for 48 h, followed by ChIP assays with a control or an anti‐phos S2 Pol II antibody. The immunoprecipitated protein was subjected to immunoblot analysis with an anti‐phos S2 Pol II antibody (G), and the immunoprecipitated DNA was examined by PCR (H) with primers targeting the *CEP55* promoter or the *CEP55* 3′‐UTR. Error bars represent standard error. * indicates *P* < 0.05, **P < 0.01, and ***P < 0.001.

As DDX21 is known to bind to target gene promoters and activates gene transcription [15], we carried out Affymetrix microarray on DOX‐inducible control or DDX21 shRNA BE(2)‐C cells after treatment with vehicle control or DOX for 32 h to examine differential gene expression. The CEP55 gene was significantly downregulated by the DDX21 shRNAs (Table S1). The results were confirmed by RT‐PCR and western blot analyses, which demonstrated that treatment with DOX in the DOX‐inducible DDX21 shRNA BE(2)‐C or Kelly cells considerably reduced DDX21 expression, and resulted in a decrease in CEP55 mRNA and protein expression (Fig. 2A,B).

To confirm that DDX21 was transcriptionally upregulating CEP55 expression, ChIP assays were carried out with a control or an anti‐DDX21 antibody in BE(2)‐C cell lysates. The DDX21 antibody effectively pulled down DDX21 protein, and binding of DDX21 was enhanced by twofold to threefold at the *CEP55* gene promoter region (Amplicon B), compared with negative control regions (Amplicon A) (Fig. 2C–E). To further demonstrate the transcriptional control of CEP55 by DDX21, a LightSwitch luciferase reporter construct containing the *CEP55* gene promoter region was transfected into the DOX‐inducible control or DDX21 shRNA BE(2)‐C cells followed by luciferase assays. Knockdown of DDX21 expression by treatment with DOX significantly downregulated the luciferase activity of the *CEP55* promoter (Fig. 2F).

The C‐terminal repeat domain of RNA polymerase II (Pol II) is phosphorylated at serine 2 (S2) near the end of genes during transcriptional elongation [26, 27, 28]. DDX21 binds to target gene promoters and regulates target gene transcriptional elongation [15]; thus, we examined whether DDX21 mediates CEP55 transcriptional elongation using ChIP assays with a control or phosphorylated S2 Pol II antibody in the DOX‐inducible DDX21 shRNA‐1 BE(2)‐C cells. The phosphorylated S2 Pol II antibody efficiently pulled down the phosphorylated S2 Pol II protein (Fig. 2G), and knocking down DDX21 significantly decreased phosphorylated S2 Pol II binding at the transcriptional termination site (3′‐untranslated region) of *CEP55* but not at the *CEP55* gene promoter (Fig. 2H). Next, we examined the transcriptional elongation modulation by DDX21 of other known DDX21 target/nontarget genes. RPLP2 is a known DDX21 target gene, while β‐actin is a known DDX21 nontarget gene [15]. Knocking down DDX21 significantly decreased the binding of phosphorylated S2 Pol II at the transcriptional termination site of *RPLP2* but not at *RPLP2* gene promoter (Fig. S1A). In contrast, knocking down DDX21 did not affect phosphorylated S2 Pol II binding to the transcriptional termination site of *β‐actin* or *β‐actin* gene promoter (Fig. S1B).

The data therefore demonstrate that DDX21 binds to the *CEP55* gene promoter to upregulate *CEP55* transcriptional elongation and expression.

### 3.3 DDX21 and CEP55 are essential for neuroblastoma cell growth

**Fig. 3:**
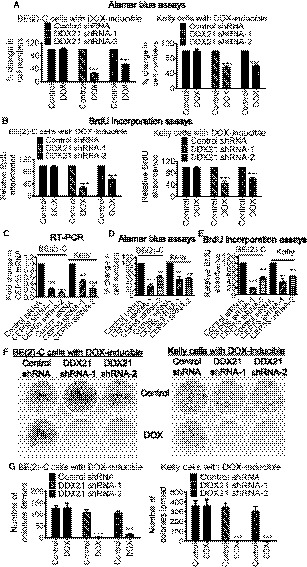
DDX21 induces neuroblastoma cell proliferation through upregulating CEP55 expression. (A–B) DOX‐inducible control shRNA, DDX21 shRNA‐1, or DDX21 shRNA‐2 BE(2)‐C and Kelly cells were treated with control or DOX for 72 h, followed by Alamar blue assays (A) or BrdU incorporation assays (B). (C‐E) BE(2)‐C and Kelly cells were transfected with control siRNA, CEP55 siRNA‐1, or CEP55 siRNA‐2 for 48 h (C) or 72 h (D, E), followed by RT‐PCR (C), Alamar blue assays (D), or BrdU incorporation assays (E). (F–G) DOX‐inducible control shRNA, DDX21 shRNA‐1, or DDX21 shRNA‐2 BE(2)‐C and Kelly cells were treated with vehicle control or DOX for 14 days. Cell colonies were fixed with methanol and stained with crystal violet (F), and the numbers of colonies were counted (G). Error bars represent standard error. * indicates *P* < 0.05, **P < 0.01, and ***P < 0.001.

We then assessed whether DDX21 or CEP55 is required for neuroblastoma cell viability and/or proliferation. Alamar blue and BrdU assays revealed that knocking down DDX21 expression in the DOX‐inducible DDX21 shRNA BE(2)‐C and Kelly cells significantly decreased cell viability (Fig. 3A) and cell proliferation (Fig. 3B) in both cell lines. We then transfected BE(2)‐C and Kelly cells with a control siRNA or two different CEP55 siRNAs. In both cell lines, the CEP55 siRNAs significantly knocked down CEP55 mRNA (Fig. 3C) and protein (Fig. S2) expression. Alamar blue and BrdU assays revealed that CEP55 knockdown also reduced cell viability (Fig. 3D) and cell proliferation (Fig. 3E) in both cell lines.

We next examined whether DDX21 increases the clonogenic capacity of neuroblastoma cells. Knocking down DDX21 almost completely abolished clonogenicity in the DOX‐inducible DDX21 shRNA‐1 and DDX21 shRNA‐2 BE(2)‐C and Kelly cells, compared with the DOX‐inducible control shRNA cells (Fig. 3F–G), indicating that DDX21 plays a crucial role in neuroblastoma tumorigenesis.

In summary, the results indicate that DDX21 and CEP55 are essential for neuroblastoma cell proliferation and survival.

### 3.4 DDX21 and CEP55 play a role in neuroblastoma cell cytoskeletal stability

**Fig. 4:**
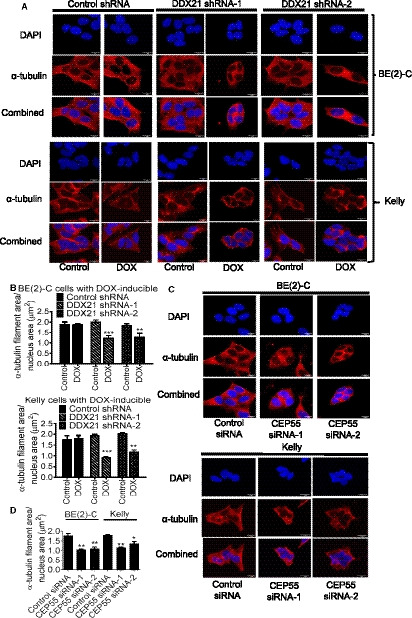
DDX21 and CEP55 play a role in neuroblastoma cell cytoskeletal stability. (A–B) DOX‐inducible control shRNA, DDX21 shRNA‐1, or DDX21 shRNA‐2 BE(2)‐C and Kelly cells were treated with vehicle control or DOX for 72 h, followed by immunostaining with an anti‐α‐tubulin antibody and counter‐staining of cell nuclei with DAPI (A). The areas covered by α‐tubulin filaments, relative to nucleus, were quantified (B). (C–D) BE(2)‐C and Kelly cells were transfected with control siRNA, CEP55 siRNA‐1, or CEP55 siRNA‐2 for 72 h followed by immunostaining with an anti‐α‐tubulin antibody and counter‐staining of cell nuclei with DAPI (C). The areas covered by α‐tubulin filaments, relative to nucleus, were quantified (D). Error bars represent standard error. * indicates *P* < 0.05, ** *P* < 0.01, and *** *P* < 0.001. Scale bar: 10 µm.

CEP55 is necessary for microtubule bundling and cytokinesis [29, 30]. Microtubules are important in cell cytoskeleton and maintenance of cellular structure [31]. We therefore examined whether DDX21 or CEP55 plays key roles in microtubule stability in neuroblastoma cells. DOX‐inducible control or DDX21 shRNA BE(2)‐C and Kelly cells were treated with vehicle control or DOX for 72 h, and stained with an anti‐α‐tubulin antibody (Fig. 4A,B) or with actin‐binding phalloidin (Fig. S3A). Knockdown of DDX21 significantly reduced the area of α‐tubulins and actin filaments. Similarly, BE(2)‐C and Kelly cells were transfected with control siRNA or two different CEP55 siRNAs and stained with an anti‐α‐tubulin antibody (Fig. 4C,D) or actin‐binding phalloidin (Fig. S3B). Knockdown of CEP55 decreased the area of α‐tubulins and actin filaments. Moreover, overexpression of CEP55 in BE(2)‐C cells containing DOX‐inducible control or DDX21 shRNA (Fig. S4) reversed the cytoskeletal damage induced by DDX21 knockdown (Fig. S5).

We then performed cell cycle analysis to determine whether the regulation of cytoskeletal stability by DDX21 and CEP55 affects cell cycle progression. Knockdown of in BE(2)‐C and Kelly cells containing DOX‐inducible DDX21 shRNAs significantly decreased the percentage of cells in S phase compared with control shRNA (Fig. S6A). Similarly, transfection of CEP55 siRNAs in BE(2)‐C and Kelly cells significantly reduced the percentage of cells in S phase compared with control siRNA (Fig. S6B).

Taken together, these data indicate that DDX21 and CEP55 play important roles in maintaining neuroblastoma cell cytoskeletal stability and in driving cell proliferation.

### 3.5 DDX21 promotes neuroblastoma progression in mice

**Fig. 5:**
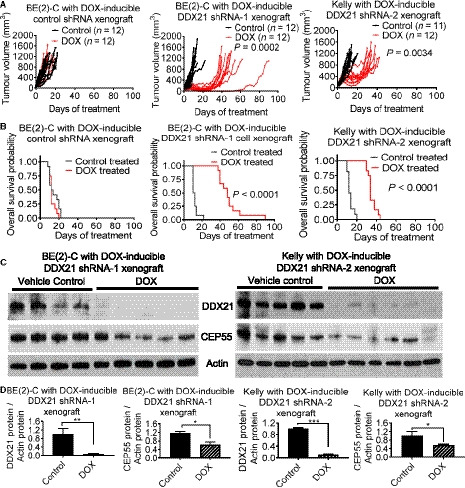
DDX21 promotes CEP55 expression and neuroblastoma progression in mice. (A–B) DOX‐inducible control shRNA BE(2)‐C, DDX21 shRNA‐1 BE(2)‐C, or DDX21 shRNA‐2 Kelly cells were xenografted into nude mice. When tumors reached 0.05 cm^3^, the three groups of mice were randomized into two subgroups each and given feed with or without DOX. Tumor growth was monitored, and the mice were culled when the tumors reached 1.0 cm^3^ or 3 months later if tumors regressed (A). Survival curves showed the probability of overall survival of the mice, and *P* values were obtained with two‐sided log‐rank test (B). (C–D) Protein was extracted from the mouse tumor tissues and subjected to western blot analysis of DDX21 and CEP55 protein expression with actin as a loading control (C). DDX21 and CEP55 protein expression was quantified, relative to actin (D). Error bars represent standard error. * indicates *P* < 0.05, ** *P* < 0.01, and *** *P* < 0.001.

We next examined whether DDX21 contributes to neuroblastoma tumor progression *in vivo*. BE(2)‐C cells containing DOX‐inducible control shRNA or DDX21 shRNA‐1 and Kelly cells containing DDX21 shRNA‐2 were xenografted into BALB/c mice. Knocking down DDX21 by treatment with DOX in mice xenografted with BE(2)‐C cells containing DOX‐inducible DDX21 shRNA‐1 or Kelly cells containing DOX‐inducible DDX21 shRNA‐2 cells led to tumor shrinkage and a significant delay in relapse, with increased overall survival by up to 68 and 24 days, respectively. No effect was observed in mice xenografted with BE(2)‐C cells containing DOX‐inducible control shRNA (Fig. 5A,B). Proteins were extracted from the tumors, and western blot analyses demonstrated that DOX treatment significantly reduced DDX21 and CEP55 protein expression in DOX‐inducible DDX21 shRNA BE(2)‐C and Kelly xenografts (Fig. 5C,D). The results therefore indicate that DDX21 regulates CEP55 expression and promotes neuroblastoma progression *in vivo*.

### 3.6 High DDX21 expression correlates with high N‐Myc and CEP55 expression and predicts poor outcome in neuroblastoma patients

**Fig. 6:**
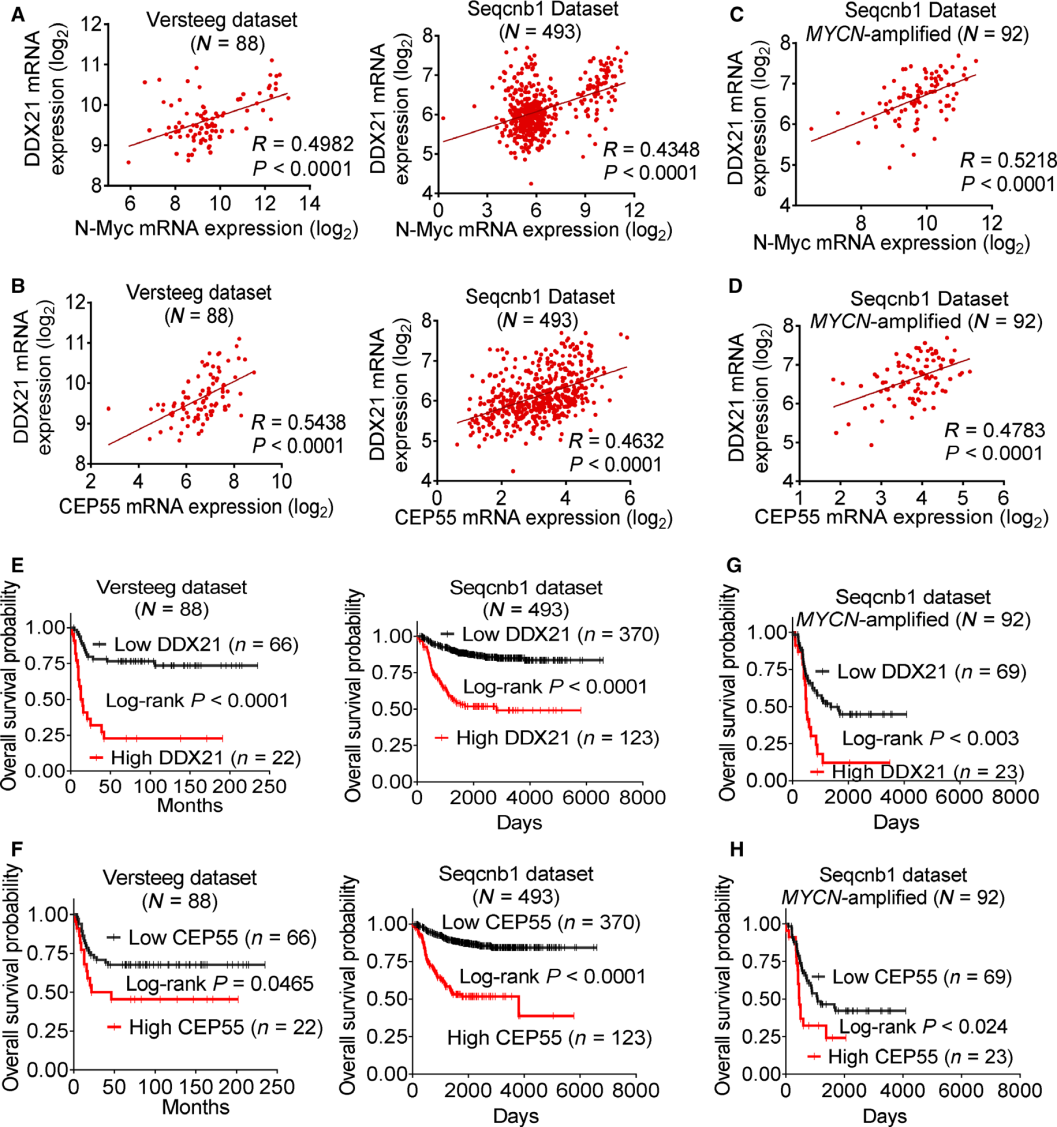
High DDX21 expression correlates with high N‐Myc and CEP55 expression and predicts poor patient prognosis. (A*–*B) Two‐sided Pearson's correlation was used to analyze the correlation between DDX21 mRNA expression and N‐Myc mRNA expression (A) or between DDX21 mRNA expression and CEP55 mRNA expression (B) in 88 or 493 human neuroblastoma tumor tissues in the publicly available microarray gene expression Versteeg dataset or RNA sequencing gene expression SEQC‐RPM‐seqcnb1 dataset, respectively, downloaded from the R2 platform (http://r2.amc.nl). Five out of 498 samples in the SEQC‐RPM‐seqcnb1 dataset were excluded from analysis, due to the lack of information on *MYCN* amplification status. (C*–*D) Two‐sided Pearson’s correlation was used to analyze the correlation between DDX21 mRNA expression and N‐Myc mRNA expression (C) or between DDX21 mRNA expression and CEP55 mRNA expression (D) in 92 *MYCN*‐amplified human neuroblastoma tissues in the RNA sequencing SEQC‐RPM‐seqcnb1 dataset. (E*–*F) Kaplan–Meier curves showed the probability of overall survival of neuroblastoma patients according to the levels of DDX21 (E) and CEP55 (F) expression in 88 and 493 human neuroblastoma tissues in the Versteeg and SEQC‐RPM‐seqcnb1 datasets, respectively, using the upper quartile of RNA expression as the cutoff point and two‐sided log‐rank test. (G*–*H) Kaplan–Meier curves showed the probability of overall survival of neuroblastoma patients according to the levels of DDX21 (G) and CEP55 (H) expression in 92 *MYCN*‐amplified neuroblastoma tissues in the large SEQC‐RPM‐seqcnb1 dataset using upper quartile of RNA expression as the cutoff point and two‐sided log‐rank test.

To investigate the clinical implications of DDX21 and CEP55 in *MYCN*‐amplified neuroblastoma, we examined DDX21 and CEP55 expression in 88 and 493 human neuroblastoma tissues in the publicly available Affymetrix microarray Versteeg dataset and RNAseq SEQC‐RPM‐seqcnb1 dataset downloaded from the R2 platform (http://r2.amc.nl) on July 7, 2017, and April 6, 2018, respectively [32, 33]. High DDX21 mRNA expression correlated with high N‐Myc mRNA expression and CEP55 mRNA expression by Pearson's correlation analysis (Fig. 6A,B). These correlations were also observed in the subset of 92 *MYCN*‐amplified patients in the SEQC‐RPM‐seqcnb1 dataset (Fig. 6C,D).

Furthermore, the Kaplan–Meier survival analysis with upper quartile as the cutoff point revealed that high DDX21 (Fig. 6E) or CEP55 (Fig. 6F) expression correlated with lower patient survival in both datasets. Consistently, in the SEQC‐RPM‐seqcnb1 dataset, high DDX21 (Fig. 6G) or CEP55 (Fig. 6H) mRNA expression in the subset of 92 *MYCN*‐amplified neuroblastoma patients also correlated with poorer patient survival. Importantly, in the larger SEQC‐RPM‐seqcnb1 dataset, multivariable Cox regression analysis with the median or upper quartile as the cutoff points revealed that high DDX21 or CEP55 expression predicted poor event‐free and overall patient survival independent of age at diagnosis, stage of disease, and *MYCN* status (Table 1).

**Table 1:** Multivariable Cox regression analysis of DDX21 and CEP55 as factors prognostic for outcome in 493 patients^a^.

Factors	Overall survival		Event‐free survival	
	HR (95% CI)	*P* value	HR (95% CI)	*P* value
High DDX21 expression (median as the cutoff)	1.99 (1.18–3.36)	0.0098	1.69 (1.20–2.38)	0.0029
*MYCN* amplification	3.07 (2.00–4.72)	< 0.0001	1.54 (1.09–2.19)	0.0145
Age > 18 months	2.38 (1.55–3.66)	< 0.0001	1.78 (1.29–2.46)	< 0.0001
Stages 3 and 4^b^	5.98 (2.86–12.48)	< 0.0001	2.81 (1.89–4.19)	0.0004
High DDX21 expression (upper quartile as the cutoff)	1.94 (1.26–2.97)	0.0025	1.52 (1.08–2.13)	0.0163
*MYCN* amplification	3.02 (1.95–4.67)	< 0.0001	1.56 (1.09–2.25)	0.0160
Age > 18 months	2.53 (1.65–3.89)	< 0.0001	1.82 (1.32–2.51)	0.0003
Stages 3 and 4^b^	5.93 (2.84–12.38)	< 0.0001	2.89 (1.94–4.31)	< 0.0001
High CEP55 expression (median as the cutoff)	3.01 (1.69–5.35)	0.0002	1.98 (1.39–2.83)	0.0002
*MYCN* amplification	3.28 (2.19–4.91)	< 0.0001	1.64 (1.17–2.28)	0.0038
Age > 18 months	2.19 (1.41–3.40)	0.0005	1.75 (1.26–2.43)	0.0009
Stages 3 and 4^b^	4.94 (2.33–10.49)	< 0.0001	2.45 (1.62–3.72)	< 0.0001
High CEP55 expression (upper quartile as the cutoff)	2.53 (1.70–3.76)	< 0.0001	2.16 (1.58–2.96)	< 0.0001
*MYCN* amplification	3.68 (2.46–5.51)	< 0.0001	1.64 (1.17–2.28)	0.0036
Age > 18 months	2.58 (1.68–3.98)	< 0.0001	1.83 (1.32–2.52)	0.0003
Stages 3 and 4^b^	5.35 (2.55–11.21)	< 0.0001	2.54 (1.69–3.82)	< 0.0001

^a^ The level of DDX21 or CEP55 expression was considered high or low in relation to the median or upper quartile level of expression in tumors of the RNA sequencing gene expression‐patient prognosis SEQC‐RPM‐seqcnb1 dataset. HRs were calculated as the antilogs of the regression coefficients in the proportional hazard regression. Multivariable Cox regression analysis was carried out following the inclusion of the four factors listed above into the Cox regression model, and *P* value was obtained from two‐sided log‐rank test.

^b^ Tumor stage was categorized as favorable (International Neuroblastoma Staging System stages 1, 2 and 4S) or unfavorable (International Neuroblastoma Staging System stages 3 and 4).

**Fig. 7:**
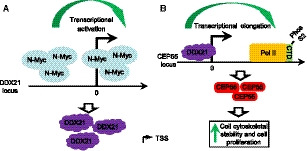
Schematic model of the regulation of DDX21 and CEP55 by N‐Myc. (A) N‐Myc binds to the *DDX21* gene promoter containing the noncanonical E‐boxes, leading to *DDX21* transcriptional activation, and increased DDX21 mRNA and protein expression. (B) DDX21 binds to the *CEP55* gene promoter to facilitate CEP55 transcriptional elongation, leading to elevated CEP55 mRNA and protein expression, as well as cell cytoskeletal stability and cell proliferation. TSS, transcription start site.

In summary, these data demonstrate that N‐Myc regulates DDX21 expression and DDX21 regulates CEP55 expression in human neuroblastoma (Fig. 7) and that high DDX21 and high CEP55 expressions are independent prognostic markers of poor outcome in human neuroblastoma patients.

## 4 Discussion

The DEAD‐box RNA helicase protein DDX21 associates with diverse species of RNA and RNA polymerase I‐ and II‐transcribed genes, facilitates the release of the positive transcription elongation factor b (P‐TEFb) from the 7SK small nuclear ribonucleoproteins in a helicase‐dependent manner, and promotes transcription of target genes [15]. DDX21 unwinds the nucleic acid structure R loops and overcomes R‐loop‐mediated stalling of RNA polymerases. By resolving estrogen‐induced R loops on estrogen‐responsive genes in breast cancer cells, DDX21 promotes transcription elongation [34]. N‐Myc and c‐Myc oncoproteins are well known to induce tumorigenesis by binding to Myc‐responsive E‐boxes at target gene promoters to drive gene transcription [5, 35]. In this study, we have identified two Myc‐responsive E‐boxes at the *DDX21* gene promoter, and our ChIP assays confirm that N‐Myc binds to the *DDX21* gene promoter. Knockdown of N‐Myc significantly decreased DDX21 mRNA and protein expression. In human neuroblastoma tissues, high levels of DDX21 expression correlate with high levels of N‐Myc expression and predict poor patient prognosis, independent of the current standard prognostic markers.

DDX21 has recently been shown to be involved in various cancers. When DDX21 is relocalized from the nucleolus to the nucleoplasm, the p53 tumor suppressor protein is activated in neural crest cells [36]. DDX21 unwinds the nucleic acid structure R loops, whereas depletion of DDX21 leads to accumulation of cellular R loops, DNA double‐strand breaks, and genomic instability [34]. DDX21 also forms a protein complex with the leukemogenic Amino‐terminal Enhancer of Split protein, and DDX21 knockdown has been shown to reduce leukemia cell proliferation and clonogenesis [18]. In addition, DDX21 is highly expressed in human breast cancer tissues and DDX21 protein expression level correlates with cancer cell proliferation rate, while mechanistically, DDX21 induces AP‐1 transcriptional activity and c‐Jun protein phosphorylation [16]. We found that DDX21 promotes *MYCN*‐amplified neuroblastoma cell proliferation and clonogenic capacity, and high DDX21 gene expression correlates with poor patient prognosis. In mouse models of *MYCN*‐amplified neuroblastoma, DDX21 knockdown initially induces tumor regression, suppresses tumor progression, and significantly improves the overall survival.

CEP55 is a centrosomal protein that has been shown to be deregulated in a number of cancers. CEP55 is upregulated when the MEK1/2‐MYC pathway is activated in breast cancer cells and is essential for breast cancer cell aneuploidy, proliferation, and survival [37], and CEP55 overexpression in primary breast tumor tissues is a marker for chromosomal instability and poor prognosis [37, 38]. CEP55 also protects aneuploid cells against cell death, and loss of CEP55 leads to caspase‐dependent apoptosis [37]. In addition, CEP55 is overexpressed in human osteosarcoma tissues, and its overexpression correlates with poor patient prognosis [39]. CEP55 knockdown significantly induces cell cycle arrest at G1 phase, suppresses osteosarcoma cell proliferation *in vitro* through regulating the PI3K/AKT pathway, and suppresses osteosarcoma tumor growth in mice [39]. CEP55 associates with microtubules, efficiently bundles microtubules, and is essential for cell proliferation [29, 30]. In the current study, genome‐wide differential gene expression analysis identifies CEP55 as one of the few genes markedly downregulated after DDX21 knockdown in neuroblastoma cells. In human neuroblastoma tumor tissues, high CEP55 gene expression correlated with high DDX21 gene expression and independently predicts poor patient survival. Knocking down DDX21 or CEP55 significantly decreases neuroblastoma cell cytoskeletal stability and cell proliferation.

## 5 Conclusions

Our data demonstrate that DDX21 and CEP55 are potential therapeutic targets for *MYCN*‐amplified neuroblastoma. Small molecule inhibitors of DDX21 could be developed with helicase targeting strategies similar to the discovery of DDX3 and DDX41 inhibitors [12, 13, 14], one of which (DDX3 inhibitor, RK‐33) is currently in a clinical trial in lung cancer patients. Studies have shown that CEP55‐derived peptides may facilitate vaccination strategies for breast cancer [40] and colorectal carcinoma [41]. Therefore, vaccination against CEP55 might be an avenue for further research in *MYCN*‐driven neuroblastoma.

## Conflict of interest

The authors declare no conflict of interest.

## Author contributions

PYL and TL designed and supervised the experiments. VP, AJH, JS, AET, JC, JG, BA, and PYL performed the experiments, collected the data, and analyzed the results. NH analyzed the Affymetrix microarray. MH, MDN, MK, MJH, JM, and TT provided intellectual input. PYL and TL wrote the manuscript with contributions from the coauthors.

## Supporting information


**Fig. S1.** DDX21 promotes RPLP2 transcriptional elongation.
**Fig. S2.** CEP55 siRNAs knock down CEP55 protein expression.
**Fig. S3.** DDX21 or CEP55 play a role in for neuroblastoma cell cytoskeletal stability.
**Fig. S4.** Transfection of CEP55 overexpression construct results in CEP55 overexpression.
**Fig. S5.** Overexpression of CEP55 rescues the phenotypes induced by DDX21 knockdown.
**Fig. S6.** DDX21 and CEP55 promote neuroblastoma cell proliferation.

**Table S1.** Genes commonly up‐ or downregulated by DDX21 shRNAs by ≥ 2 fold.

## Data Availability

The Affymetrix Microarray data supporting the conclusions of this article are available at the NCBI GEO database website, with accession number GSE127994. The publicly available microarray gene expression Versteeg dataset or RNA sequencing gene expression SEQC‐RPM‐seqcnb1 dataset used in this study were downloaded from the R2 platform (http://r2.amc.nl).

## References

[1] Maris JM , Hogarty MD , Bagatell R & Cohn SL (2007) Neuroblastoma. Lancet 369, 2106–2120. 17586306 10.1016/S0140-6736(07)60983-0

[2] Matthay KK , Maris JM , Schleiermacher G , Nakagawara A , Mackall CL , Diller L & Weiss WA (2016) Neuroblastoma. Nat Rev Dis Primers 2, 16078. 27830764 10.1038/nrdp.2016.78

[3] Weiss WA , Aldape K , Mohapatra G , Feuerstein BG & Bishop JM (1997) Targeted expression of MYCN causes neuroblastoma in transgenic mice. EMBO J 16, 2985–2995. 9214616 10.1093/emboj/16.11.2985 PMC1169917

[4] Dang CV (2013) MYC on the path to cancer. Cell 149, 22–35. 10.1016/j.cell.2012.03.003 PMC3345192 22464321

[5] Eilers M & Eisenman RN (2008) Myc's broad reach. Genes Dev 22, 2755–2766. 18923074 10.1101/gad.1712408 PMC2751281

[6] Soucek L & Evan GI (2010) The ups and downs of Myc biology. Curr Opin Genet Dev 20, 91. 19962879 10.1016/j.gde.2009.11.001 PMC2822095

[7] Beltran H (2014) The N‐myc oncogene: maximizing its targets, regulation, and therapeutic potential. Mol Cancer Res 12, 815–822. 24589438 10.1158/1541-7786.MCR-13-0536

[8] Sloan KE & Bohnsack MT (2018) Unravelling the mechanisms of RNA helicase regulation. Trends Biochem Sci 43, 237–250. 29486979 10.1016/j.tibs.2018.02.001

[9] Cai W , Chen Z , Rane G , Singh S , Choo Z , Wang C , Yuan Y , Tan T , Arfuso F , Yap C *et al*. (2017) Wanted DEAD/H or alive: helicases winding up in cancers. J Natl Cancer Inst 109, djw278. 10.1093/jnci/djw278 28122908

[10] Perčulija V & Ouyang S (2019) Diverse roles of DEAD/DEAH‐Box helicases in innate immunity and diseases. In Helicases from All Domains of Life ( Tuteja R , ed), pp. 141–171. Academic Press, Cambridge, MA.

[11] Bordeleau ME , Cencic R , Lindqvist L , Oberer M , Northcote P , Wagner G & Pelletier J (2006) RNA‐mediated sequestration of the RNA helicase eIF4A by Pateamine A inhibits translation initiation. Chem Biol 13, 1287–1295. 17185224 10.1016/j.chembiol.2006.10.005

[12] Bol GM , Vesuna F , Xie M , Zeng J , Aziz K , Gandhi N , Levine A , Irving A , Korz D , Tantravedi S *et al*. (2015) Targeting DDX3 with a small molecule inhibitor for lung cancer therapy. EMBO Mol Med 7, 648–669. 25820276 10.15252/emmm.201404368 PMC4492822

[13] Radi M , Falchi F , Garbelli A , Samuele A , Bernardo V , Paolucci S , Baldanti F , Schenone S , Manetti F , Maga G *et al*. (2012) Discovery of the first small molecule inhibitor of human DDX3 specifically designed to target the RNA binding site: towards the next generation HIV‐1 inhibitors. Bioorg Med Chem Lett 22, 2094–2098. 22300661 10.1016/j.bmcl.2011.12.135

[14] Yoneyama‐Hirozane M , Kondo M , Matsumoto SI , Morikawa‐Oki A , Morishita D , Nakanishi A , Kawamoto T & Nakayama M (2017) High‐throughput screening to identify inhibitors of DEAD box helicase DDX41. SLAS Discov 22, 1084–1092. 28426938 10.1177/2472555217705952

[15] Calo E , Flynn RA , Martin L , Spitale RC , Chang HY & Wysocka J (2015) RNA helicase DDX21 coordinates transcription and ribosomal RNA processing. Nature 518, 249–253. 25470060 10.1038/nature13923 PMC4827702

[16] Zhang Y , Baysac KC , Yee LF , Saporita AJ & Weber JD (2014) Elevated DDX21 regulates c‐Jun activity and rRNA processing in human breast cancers. Breast Cancer Res 16, 449. 25260534 10.1186/s13058-014-0449-z PMC4303128

[17] Jung Y , Lee S , Choi HS , Kim SN , Lee E , Shin Y , Seo J , Kim B , Kim WK , Chun HK *et al*. (2011) Clinical validation of colorectal cancer biomarkers identified from bioinformatics analysis of public expression data. Clin Cancer Res 17, 700–709. 21304002 10.1158/1078-0432.CCR-10-1300

[18] Zhou F , Liu Y , Rohde C , Pauli C , Gerloff D , Kohn M , Misiak D , Baumer N , Cui C , Gollner S *et al*. (2017) AML1‐ETO requires enhanced C/D box snoRNA/RNP formation to induce self‐renewal and leukaemia. Nat Cell Biol 19, 844–855. 28650479 10.1038/ncb3563

[19] Tee A , Ciampa O , Wong M , Fletcher J , Kamili A , Chen J , Ho N , Sun Y , Carter D , Cheung B *et al*. (2020) Combination therapy with the CDK7 inhibitor and the tyrosine kinase inhibitor exerts synergistic anticancer effects against MYCN‐amplified neuroblastoma. Int J Cancer 147, 1928–1938. 32086952 10.1002/ijc.32936

[20] Herold MJ , van den Brandt J , Seibler J & Reichardt HM (2008) Inducible and reversible gene silencing by stable integration of an shRNA‐encoding lentivirus in transgenic rats. Proc Natl Acad Sci USA 105, 18507–18512. 19017805 10.1073/pnas.0806213105 PMC2587564

[21] Liu PY , Tee AE , Milazzo G , Hannan KM , Maag J , Mondal S , Atmadibrata B , Bartonicek N , Peng H , Ho N *et al*. (2019) The long noncoding RNA lncNB1 promotes tumorigenesis by interacting with ribosomal protein RPL35. Nat Commun 10, 5026. 31690716 10.1038/s41467-019-12971-3 PMC6831662

[22] Livak KJ & Schmittgen TD (2001) Analysis of relative gene expression data using real‐time quantitative PCR and the 2(‐Delta Delta C(T)) Method. Methods 25, 402–408. 11846609 10.1006/meth.2001.1262

[23] Liu PY , Erriquez D , Marshall GM , Tee AE , Polly P , Wong M , Liu B , Bell JL , Zhang XD , Milazzo G *et al*. (2014) Effects of a novel long noncoding RNA, lncUSMycN, on N‐Myc expression and neuroblastoma progression. J Natl Cancer Inst 106, dju113. 24906397 10.1093/jnci/dju113

[24] Pandzic E , Gelissen I , Whan R , Barter P , Sviridov D , Gaus K , Rye K & Cochran B (2017) The ATP binding cassette transporter, ABCG1, localizes to cortical actin filaments. Sci Rep 7, 42025. 28165022 10.1038/srep42025 PMC5292732

[25] Grandori C , Cowley SM , James LP & Eisenman RN (2000) The Myc/Max/Mad network and the transcriptional control of cell behavior. Annu Rev Cell Dev Biol 16, 653–699. 11031250 10.1146/annurev.cellbio.16.1.653

[26] Komarnitsky P , Cho EJ & Buratowski S (2000) Different phosphorylated forms of RNA polymerase II and associated mRNA processing factors during transcription. Genes Dev 14, 2452–2460. 11018013 10.1101/gad.824700 PMC316976

[27] Morris DP , Michelotti GA & Schwinn DA (2005) Evidence that phosphorylation of the RNA polymerase II carboxyl‐terminal repeats is similar in yeast and humans. J Biol Chem 280, 31368–31377. 16012166 10.1074/jbc.M501546200 PMC2277102

[28] Phatnani H & Greenleaf A (2006) Phosphorylation and functions of the RNA polymerase II CTD. Genes Dev 20, 2922–2936. 17079683 10.1101/gad.1477006

[29] Fabbro M , Zhou BB , Takahashi M , Sarcevic B , Lal P , Graham ME , Gabrielli BG , Robinson PJ , Nigg EA , Ono Y *et al*. (2005) Cdk1/Erk2‐ and Plk1‐dependent phosphorylation of a centrosome protein, Cep55, is required for its recruitment to midbody and cytokinesis. Dev Cell 9, 477–488. 16198290 10.1016/j.devcel.2005.09.003

[30] Zhao W , Seki A & Fang G (2006) Cep55, a microtubule‐bundling protein, associates with centralspindlin to control the midbody integrity and cell abscission during cytokinesis. Mol Biol Cell 17, 3881–3896. 16790497 10.1091/mbc.E06-01-0015 PMC1593165

[31] Fletcher DA & Mullins RD (2010) Cell mechanics and the cytoskeleton. Nature 463, 485–492. 20110992 10.1038/nature08908 PMC2851742

[32] Zhang W , Yu Y , Hertwig F , Thierry‐Mieg J , Zhang W , Thierry‐Mieg D , Wang J , Furlanello C , Devanarayan V , Cheng J *et al*. (2015) Comparison of RNA‐seq and microarray‐based models for clinical endpoint prediction. Genome Biol 16, 133. 26109056 10.1186/s13059-015-0694-1 PMC4506430

[33] Molenaar JJ , Koster J , Zwijnenburg DA , van Sluis P , Valentijn LJ , van der Ploeg I , Hamdi M , van Nes J , Westerman BA , van Arkel J *et al*. (2012) Sequencing of neuroblastoma identifies chromothripsis and defects in neuritogenesis genes. Nature 483, 589–593. 22367537 10.1038/nature10910

[34] Song C , Hotz‐Wagenblatt A , Voit R & Grummt I (2017) SIRT7 and the DEAD‐box helicase DDX21 cooperate to resolve genomic R loops and safeguard genome stability. Genes Dev 31, 1370–1381. 28790157 10.1101/gad.300624.117 PMC5580657

[35] Dang Chi V (2012) MYC on the path to cancer. Cell 149, 22–35. 22464321 10.1016/j.cell.2012.03.003 PMC3345192

[36] Calo E , Gu B , Bowen ME , Aryan F , Zalc A , Liang J , Flynn RA , Swigut T , Chang HY , Attardi LD *et al*. (2018) Tissue‐selective effects of nucleolar stress and rDNA damage in developmental disorders. Nature 554, 112–117. 29364875 10.1038/nature25449 PMC5927778

[37] Kalimutho M , Sinha D , Jeffery J , Nones K , Srihari S , Fernando WC , Duijf PH , Vennin C , Raninga P , Nanayakkara D *et al*. (2018) CEP55 is a determinant of cell fate during perturbed mitosis in breast cancer. EMBO Mol Med 10, e8566. 30108112 10.15252/emmm.201708566 PMC6127888

[38] Carter SL , Eklund AC , Kohane IS , Harris LN & Szallasi Z (2006) A signature of chromosomal instability inferred from gene expression profiles predicts clinical outcome in multiple human cancers. Nat Genet 38, 1043–1048. 16921376 10.1038/ng1861

[39] Xu L , Xia C , Sheng F , Sun Q , Xiong J & Wang S (2018) CEP55 promotes the proliferation and invasion of tumour cells via the AKT signalling pathway in osteosarcoma. Carcinogenesis 39, 623–631. 29579156 10.1093/carcin/bgy017

[40] Inoda S , Hirohashi Y , Torigoe T , Nakatsugawa M , Kiriyama K , Nakazawa E , Harada K , Takasu H , Tamura Y , Kamiguchi K *et al*. (2009) Cep55/c10orf3, a tumor antigen derived from a centrosome residing protein in breast carcinoma. J Immunother 32, 474–485. 19609239 10.1097/CJI.0b013e3181a1d109

[41] Inoda S , Morita R , Hirohashi Y , Torigoe T , Asanuma H , Nakazawa E , Nakatsugawa M , Tamura Y , Kamiguchi K , Tsuruma T *et al*. (2011) The feasibility of Cep55/c10orf3 derived peptide vaccine therapy for colorectal carcinoma. Exp Mol Pathol 90, 55–60. 20950610 10.1016/j.yexmp.2010.10.001

