# Supplementary material for: The RNA‐helicase DDX21 upregulates CEP55 expression and promotes neuroblastoma
Source: Mol Oncol. 2021 Feb 26;15(4):1162–79. doi: 10.1002/1878-0261.12906 (PMC8024731; doi:10.1002/1878-0261.12906)
Supplement: Supplementary file 1 — Fig. S1. DDX21 promotes RPLP2 transcriptional elongation. Fig. S2. CEP55 siRNAs knock down CEP55 protein expression. Fig. S3. DDX21 or CEP55 play a role in for neuroblastoma cell cytoskeletal stability. Fig. S4. Transfection of CEP55 overexpression construct results in CEP55 overexpression. Fig. S5. Overexpression of CEP55 rescues the phenotypes induced by DDX21 knockdown. Fig. S6. DDX21 and CEP55 promote neuroblastoma cell proliferation. [file MOL2-15-1162-s001.pdf]

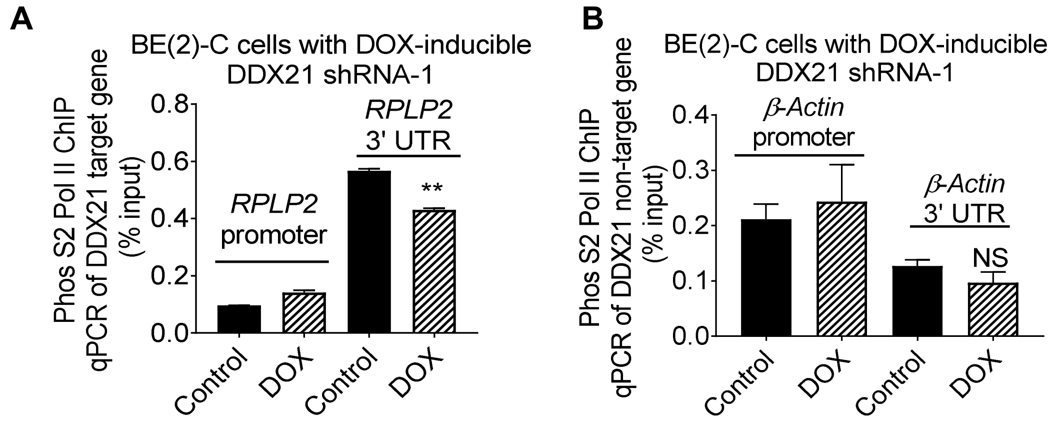

Supplementary Figure S1. DDX21 promotes RPLP2 transcriptional elongation. (A-B) BE(2)-C cells with DOX-inducible DDX21 shRNA-1 were treated with vehicle control or DOX for 48 hours, followed by ChIP assays with a control or an anti-phos S2 Pol II antibody. The immunoprecipitated DNA was examined by PCR with primers targeting the *RPLP2* or *β-actin* promoter, and the *RPLP2* or *β-actin* 3′-untranslated region (3′-UTR). Error bars represented standard error. ** indicated $P < 0.01$. NS indicated not significant.

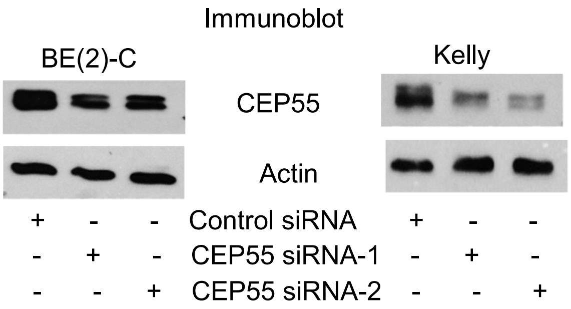

Supplementary Figure S2. CEP55 siRNAs knock down CEP55 protein expression. BE(2)-C and Kelly cells were transfected with control siRNA, CEP55 siRNA-1 or CEP55 siRNA-2 for 48 hours, followed by western blot analysis of CEP55 expression.

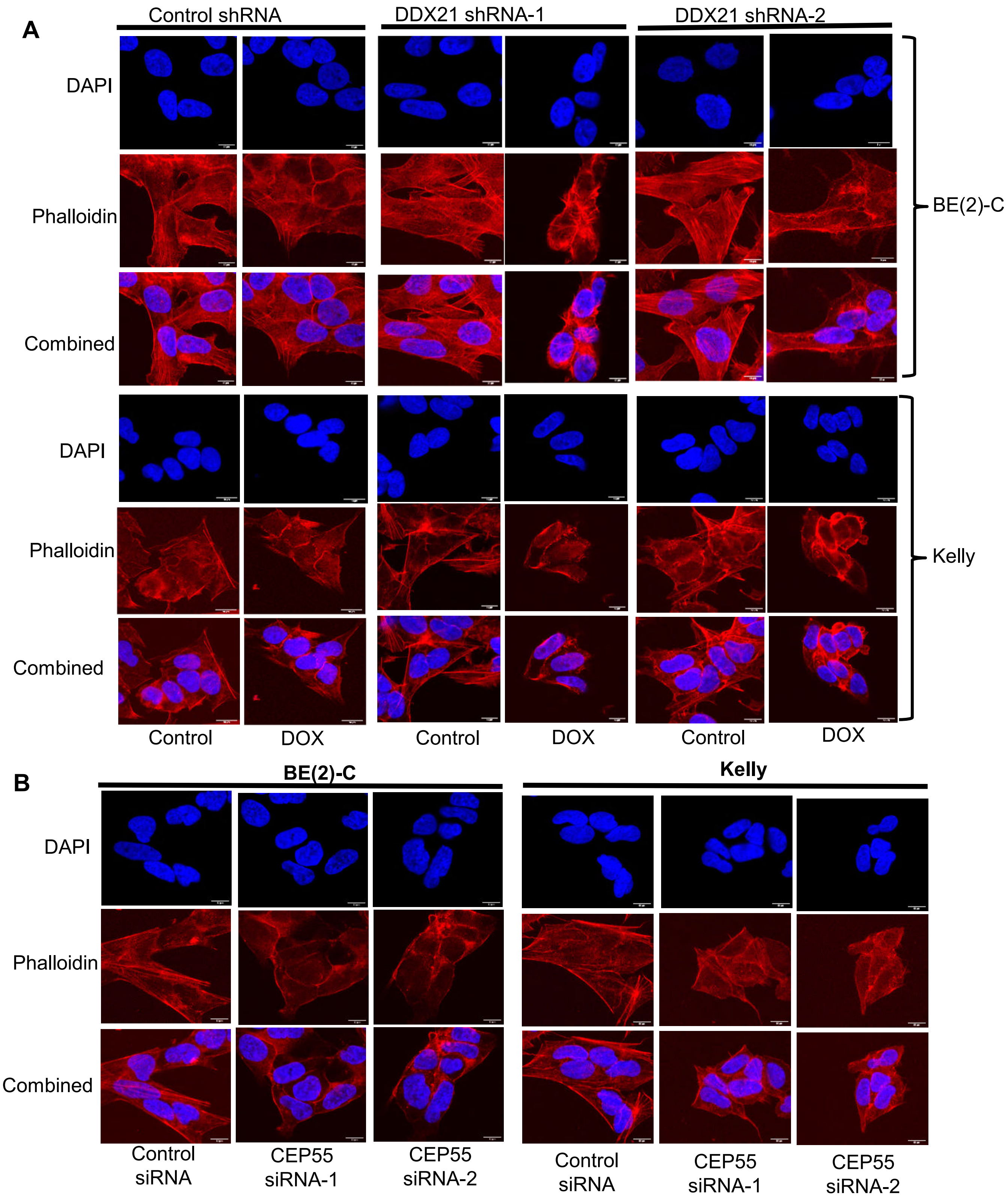

Supplementary Figure S3. DDX21 and CEP55 play a role in neuroblastoma cell cytoskeletal stability. (A) Doxycycline (DOX)-inducible control shRNA, DDX21 shRNA-1 or DDX21 shRNA-2 BE(2)-C and Kelly cells were treated with vehicle control or DOX for 72 hours, followed by immunostaining of phalloidin. DNA was counterstained with DAPI. (B) BE(2)-C and Kelly cells were transfected with control siRNA, CEP55 siRNA-1 or CEP55 siRNA-2 for 72 hours, followed by immunostaining of phalloidin. DNA was counterstained with DAPI. Scale bar: 10 μm.

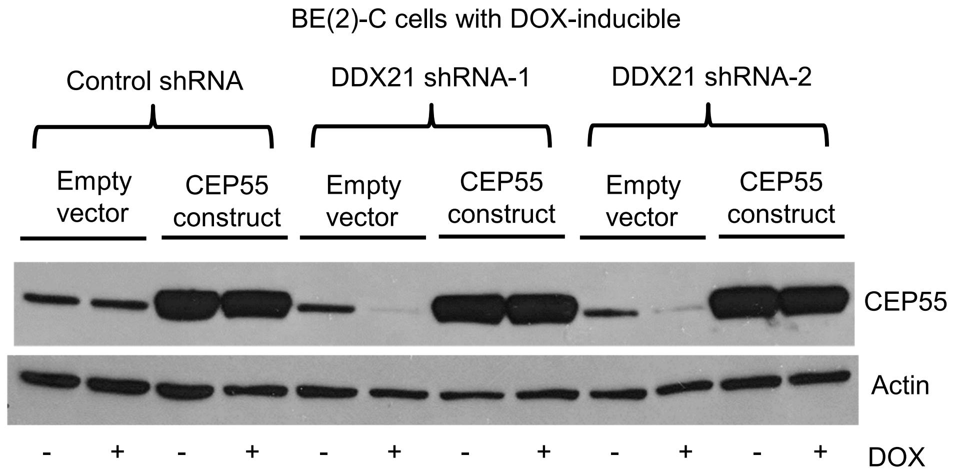

Supplementary Figure S4. Transfection of CEP55 over-expression construct results in CEP55 over-expression. DOX-inducible control shRNA, DDX21 shRNA-1 or DDX21 shRNA-2 BE(2)-C cells were transfected with an empty vector or CEP55 over-expression construct, then treated with vehicle control or DOX for 48 hours, followed by western blot analysis of CEP55 expression.

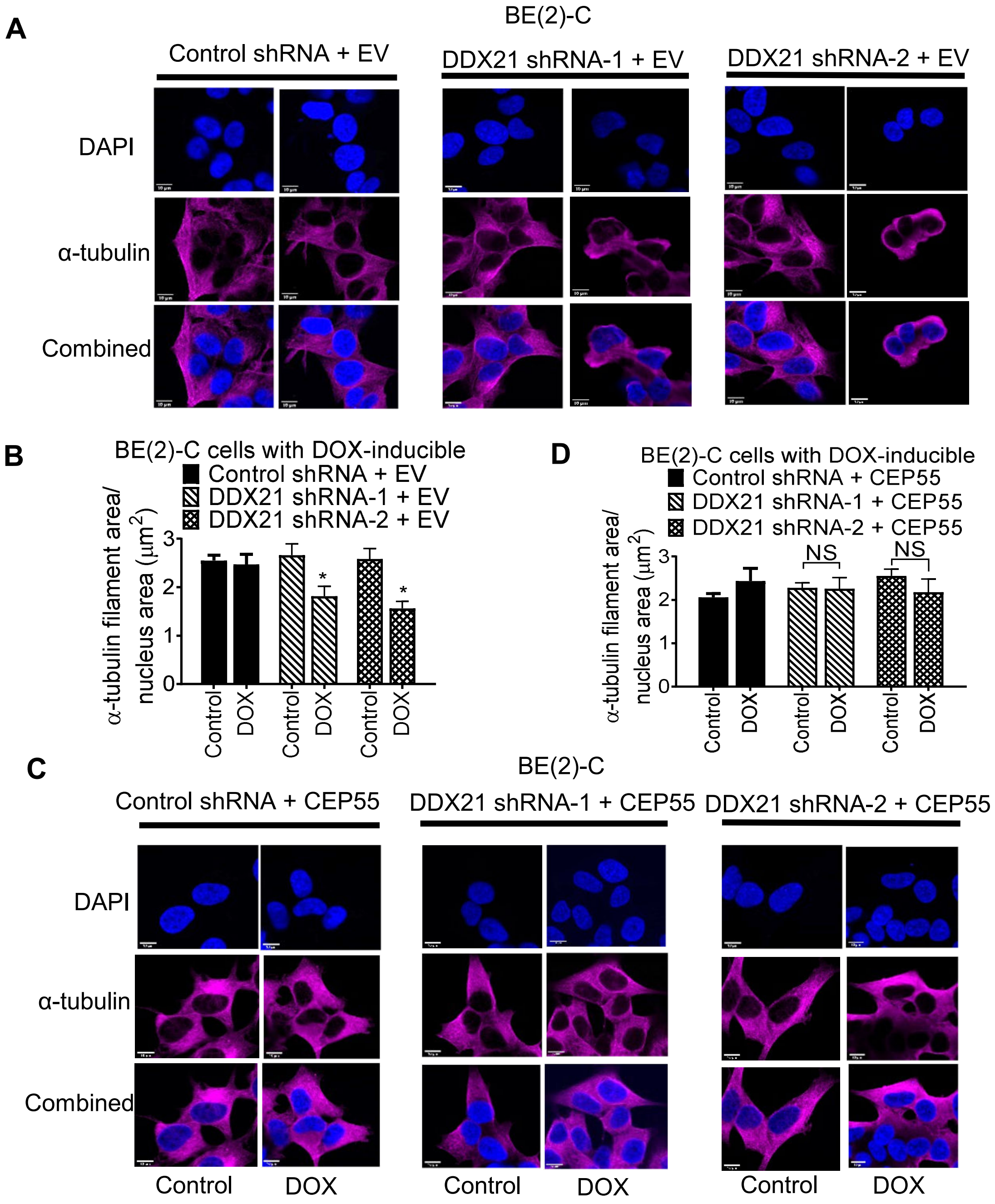

Supplementary Figure S5. Over-expression of CEP55 rescues the phenotypes induced by DDX21 knockdown. (A-B) DOX-inducible control shRNA, DDX21 shRNA-1 or DDX21 shRNA-2 BE(2)-C cells were transfected with an empty vector (EV) construct, then treated with vehicle control or DOX for 72 hours, followed by immunostaining with an anti-α-tubulin antibody and DNA was counterstained with DAPI (A). The areas covered by α-tubulin filaments, relative to nucleus, were quantified (B). (C-D) DOX-inducible control shRNA, DDX21 shRNA-1 or DDX21 shRNA-2 BE(2)-C cells were transfected with a CEP55 over-expression construct, then treated with vehicle control or DOX for 72 hours, followed by immunostaining an anti-α-tubulin antibody and DNA was counterstained with DAPI (C). The areas covered by α-tubulin filaments, relative to nucleus, were quantified (D). Error bars represented standard error. * indicated $P < 0.05$. Scale bar: 10 μm. NS indicated not significant.

A

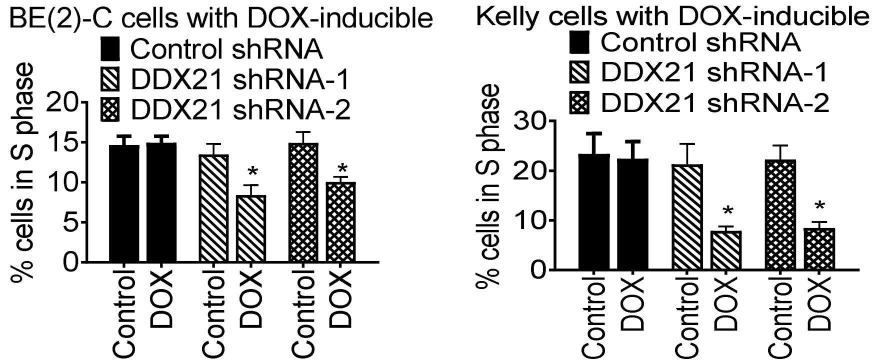

B

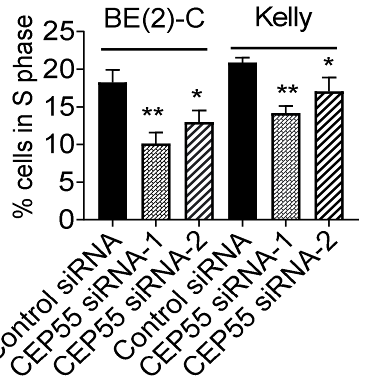

Supplementary Figure S6. DDX21 and CEP55 promote neuroblastoma cell proliferation. (A) DOX-inducible control shRNA, DDX21 shRNA-1 or DDX21 shRNA-2 BE(2)-C and Kelly cells were treated with vehicle control or DOX for 72 h, followed by staining with propidium iodide and cell cycle analysis by flow cytometry. (B) BE(2)-C and Kelly cells were transfected with control siRNA, CEP55 siRNA-1 or CEP55 siRNA-2 for 72 hours, followed by staining with propidium iodide and cell cycle analysis by flow cytometry. The percentage of cells in S phase was shown. Error bars represented standard error. * indicated $P < 0.05$ and ** $P < 0.01$.
