# Supplementary material for: The RNA‐helicase DDX21 upregulates CEP55 expression and promotes neuroblastoma
Source: Mol Oncol. 2021 Feb 26;15(4):1162–79. doi: 10.1002/1878-0261.12906 (PMC8024731; doi:10.1002/1878-0261.12906)
Supplement: Supplementary file 2 [file MOL2-15-1162-s002.docx]

Supplementary Table S1. Genes commonly up- or down-regulated by DDX21 shRNA-1 and DDX21 shRNA-2 by ≥ 2 fold compared with control shRNA, as identified by Affymetrix microarray analysis in DOX-inducible control shRNA, DDX21 shRNA-1 and DDX21 shRNA-2 BE(2)-C cells after treatment with vehicle control or DOX for 32 hours.

FC_Control shRNA_DOXvs DMSO	FC_DDX21 shRNA-1_DOXvsDMSO	FC_DDX21 shRNA-2_DOXvsDMSO	FC_DDX21 shRNAs_DOXvs DMSO	FC_DDX21 shRNAs_DOXvs DMSO_NormBy Control shRNA	Gene.Symbol
1.051092764	-4.793481951	-3.06351518	-3.832088819	-4.02788083	DDX21
1.986498908	-1.529615449	-1.825280263	-1.670920971	-3.319282683	RNU6-1255P
1.235703827	-1.895130241	-3.499328247	-2.575205387	-3.182191152	SNORA2A
1.460387272	-1.593194053	-2.009534018	-1.789295294	-2.613064072	OR2G2
1.203032819	-2.045130635	-2.169193872	-2.106248998	-2.533886669	RP11-385E5.5
1.039988085	-2.580709943	-2.183063356	-2.373573953	-2.46848863	TRBV6-4
-1.266743126	-2.439675046	-3.762967946	-3.029920625	-2.391898218	RNY4P17
1.389355698	-1.932743251	-1.502863521	-1.704303179	-2.367883333	HCG24
1.215870855	-1.754796824	-2.127020592	-1.931965056	-2.349020005	RNU4-29P
1.432237752	-1.647630799	-1.604807218	-1.626078042	-2.328930359	RNU7-96P
1.338518427	-1.696832313	-1.777665647	-1.736778775	-2.324710393	POPDC2
1.204547261	-1.857898903	-1.925657331	-1.891474727	-2.278370703	GOLGA6L7P
1.098935719	-1.789968253	-2.21063218	-1.989211256	-2.186015302	CEP55
-1.025435678	-2.410547737	-1.94128105	-2.163226905	-2.109568596	FAM111B
1.179978031	-1.517523649	-2.059117276	-1.767698833	-2.085845789	AC009499.2
1.264946177	-1.644310781	-1.621308915	-1.632769343	-2.065365339	RFPL4AL1
1.141015983	-1.698683413	-1.861749539	-1.778348408	-2.029123956	IGBP1P1
-1.013912392	-2.769746615	-1.524692173	-2.05499659	-2.026798969	RNU7-40P
1.006100204	-2.078766911	-1.886851652	-1.980485996	-1.992567365	CENPU (MLF1IP)
1.22316667	-1.512497754	-1.751865481	-1.627787641	-1.991055588	RP11-456O19.2
-2.205838243	2.267980129	1.779320007	2.008846042	4.431189423	MAP3K8
-2.438931096	1.701581201	1.661940808	1.681644207	4.101414347	RP11-589M4.3
-1.681264838	1.684550875	1.959993677	1.817060556	3.054960021	FAM45A
-1.785067959	1.725401362	1.623568213	1.673710491	2.98768697	RNU6-47P
-1.439124095	2.74680977	1.510264251	2.036764247	2.931156503	RNU1-70P
-1.470031429	2.152926399	1.509824644	1.802925771	2.650357547	CELA2B
-1.105313792	2.567001253	2.102223414	2.323017464	2.567663241	RNU6-1143P
-1.504008166	1.817514533	1.5294543	1.667274848	2.507594986	RNU6-750P
-1.265379387	2.001218924	1.651449667	1.817941783	2.300386059	RP11-555M1.3
-1.391471153	1.571907147	1.625839676	1.598645992	2.224469781	RP11-39E3.3
-1.325105292	1.523737093	1.681489768	1.600671212	2.121057895	TRGV3
-1.096994278	1.640375385	2.19622064	1.898058555	2.082159373	LCA5
-1.17037836	1.966231239	1.600874264	1.774172761	2.076453407	SESN2
-1.190213181	1.933954697	1.523235493	1.716353237	2.042826246	RNU6-519P
-1.104273856	1.700112246	1.947553554	1.819631734	2.009371751	USP6 // USP32
